# Supplementary material for: Morin Sensitizes MDA-MB-231 Triple-Negative Breast Cancer Cells to Doxorubicin Cytotoxicity by Suppressing FOXM1 and Attenuating EGFR/STAT3 Signaling Pathways
Source: Pharmaceuticals (Basel). 2023 Apr 29;16(5):672. doi: 10.3390/ph16050672 (PMC10222377; doi:10.3390/ph16050672)
Supplement: Supplementary file 1 [file pharmaceuticals-16-00672-s001.zip › pharmaceuticals-2330865-supplementary.pdf]

Supplementary materials

Morin sensitizes MDA-MB-231 triple-negative breast cancer cells to doxorubicin cytotoxicity by suppressing FOXM1 and attenuating EGFR/STAT3 signaling pathways

Sushma Maharjan, Min-Gu Lee, Soyoung Kim, Kyu-Shik Lee*, Kyung-Soo Nam*

Department of Pharmacology, College of Medicine and Intractable Disease Research Center, Dongguk University, Gyeongju, 38066, Republic of Korea

***Correspondences:**

1. namks@dongguk.ac.kr (Kyung-Soo Nam)

2. there1@dongguk.ac.kr (Kyu-Shik Lee)

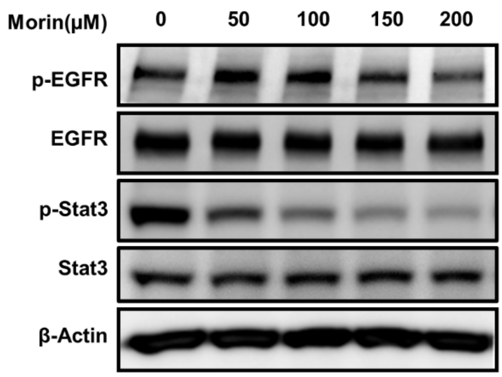

Figure S1. Morin decreased the expression of p-EGFR and p-STAT3 in MDA-MB-231 cells.
